# Supplementary material for: Antigen footprint governs activation of the B cell receptor
Source: Nat Commun. 2023 Feb 22;14:976. doi: 10.1038/s41467-023-36672-0 (PMC9947222; doi:10.1038/s41467-023-36672-0)
Supplement: Supplementary file 1 — Supplementary Information [file 41467_2023_36672_MOESM1_ESM.pdf]

Supplementary Information

Antigen footprint governs activation of the B cell receptor

Ferapontov et al. 2023

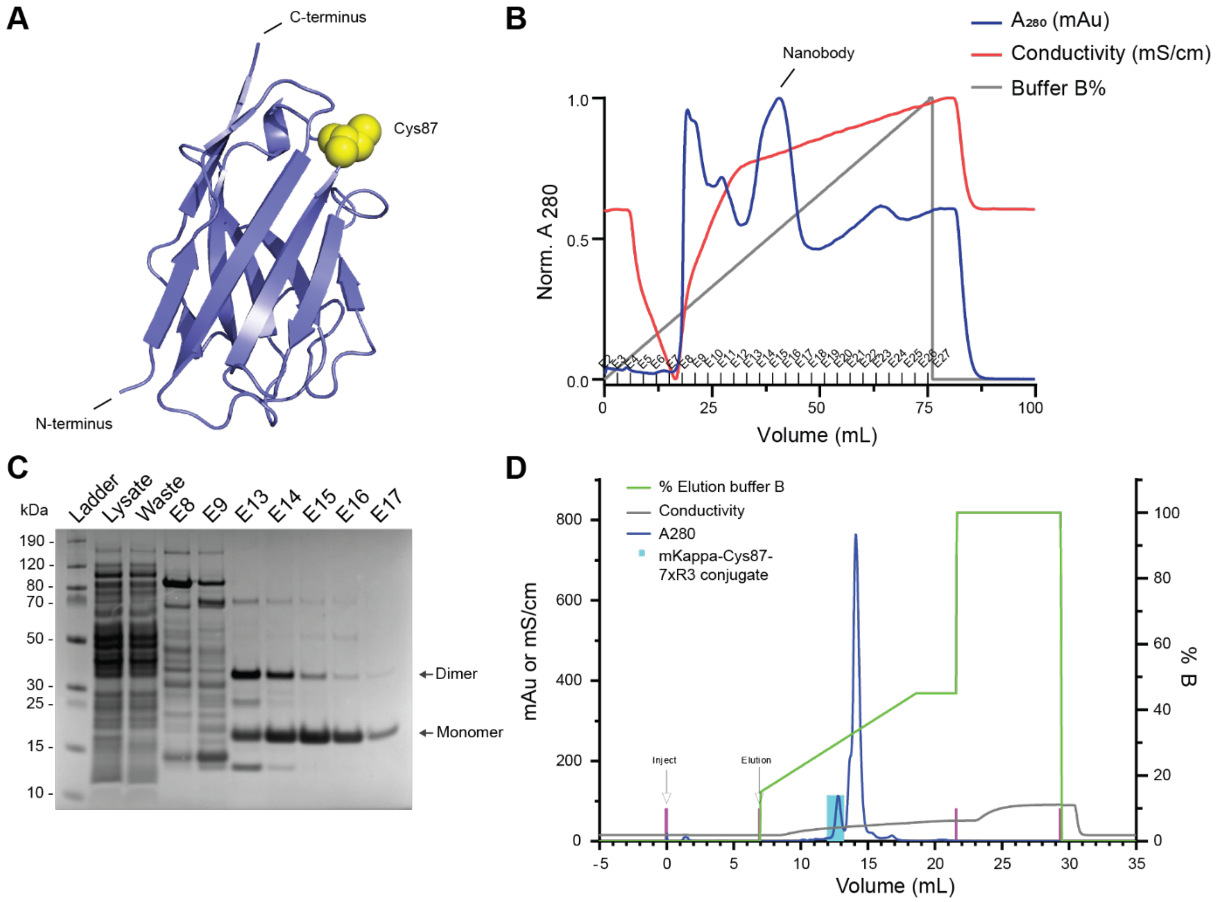

Supplementary Figure 1. Nanobody expression and purification. (A) The anti-mouse kappa LC nanobody (κLC-Nb-7xR3) was expressed with a C-terminal cysteine residue by site-directed mutagenesis of Ser87 to Cys87 for subsequent site-specific conjugation. (**B**) Äkta FPLC chromatogram of nanobody elution profile. To avoid formation of disulphide bonds between the C-terminal free cysteine residues, the nanobody was eluted with 5 mM β-mercaptoethanol (BME) in the elution buffer. (**C**) SDS-PAGE gel (4-12% Bis-tris) of the different nanobody fractions, from left: protein ladder, lysate, flow through (waste), eluted fractions E8-E17 corresponding to before and after nanobody peak. Despite the use of reducing agent, we still observed some dimer formation of the nanobody. Fractions E15-17 were pooled together for further work on the nanobody. The ladder is a PageRuler pre-stained protein ladder (10-180 kDa). The gel was stained with Coomassie Brilliant blue. (**D**) FPLC chromatogram for the purification of the κLC-Nb-7xR3 (mKappa-Cys87-7xR3) conjugate used for subsequent imaging experiments. FPLC, fast protein liquid chromatography. Source data are provided as Source Data file.

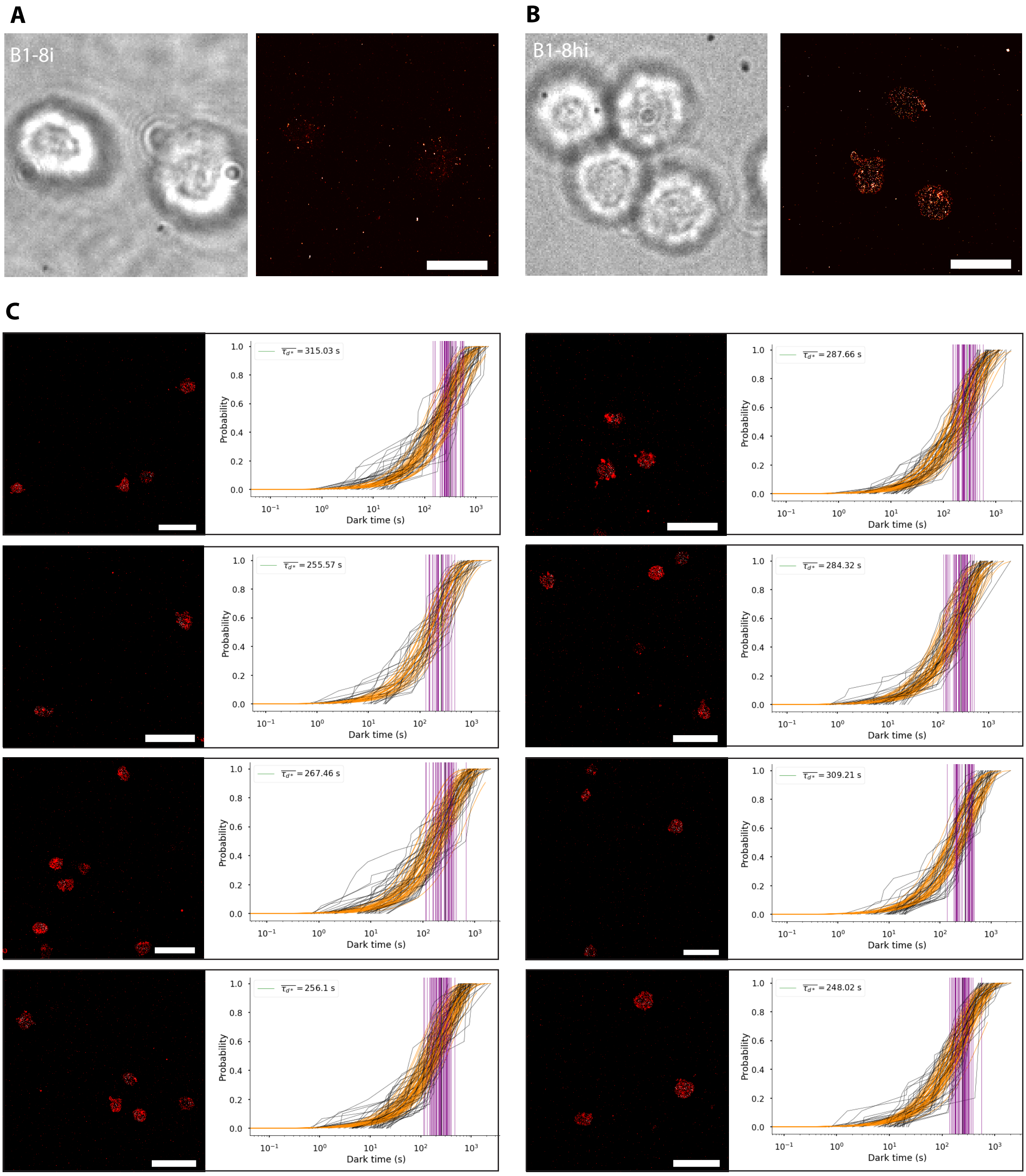

Supplementary Figure 2. Dark time fits and filtering for DNA-PAINT imaging of B1-8hi B cells with anti-kappa light chain nanobody. (**A**) Negative control for the anti-mouse kappa-light-chain nanobody-7xR3. B1-8i kappa knock-out cells are >90% kappa-light chain-negative and show low signal. (**B**) Positive control for the anti-mouse κLC-Nb-7xR3. B1-8hi B cells are >80% kappa-light chain positive and show strong signal compared to the negative control. (**C**) Images used for data analysis. Each box represents one of the 8 FOVs that were used for data analysis. Left: FOV with 2-7 cells with DNA-PAINT data (red) and picked single

binding sites (SBS, white). Right: Traces of each picked SBS were analyzed and the dark times (black) were fitted as a cumulative distribution function (orange). The mean over all individual dark times (violet) was used as the dark time corresponding to SBSs (upper left box). Scale bars A and B: 5 μm, C: 10 μm. Data are representative of 3 independent experiments and n=31 cells. Source data are provided as Source Data file.

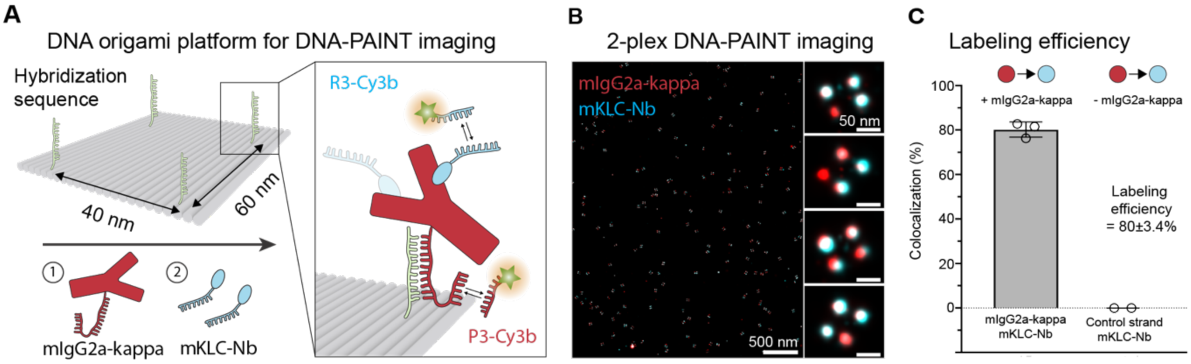

Supplementary Figure 3. Quantification of labeling efficiency for the anti-mouse kappa light chain nanobody. (A) Design of a DNA origami structure with four S1HP3H extensions for specific hybridization, spaced 40 and 60 nm apart. Single mouse IgG2a (mIgG2a) antibodies were used to mimic the kappa light chain of the B cell receptor and hybridized to the S1HP3H extensions via a covalently linked hybridization strand (S1), carrying an additional P3 handle for DNA-PAINT imaging (S1-P3). The anti-mouse kappa light chain nanobody (κLC-Nb), conjugated with 7xR3 was incubated with the DNA origami platforms carrying the mIgG2a. **(B)** The κLC-Nb as well as the mIgG2a were imaged in 2 consecutive rounds with R3-Cy3b and P3-Cy3b imagers, respectively, and show colocalization. **(C)** Labeling efficiency was measured by dividing the mIgG2a-κLC-Nb colocalization spots by the total number of mIgG2a molecules on the surface and was measured to be 80±3%. Mean ± SD is reported from a single experiment with n=3 replicates and is based on the analysis of 1000 individual structures. Source data are provided as Source Data file.

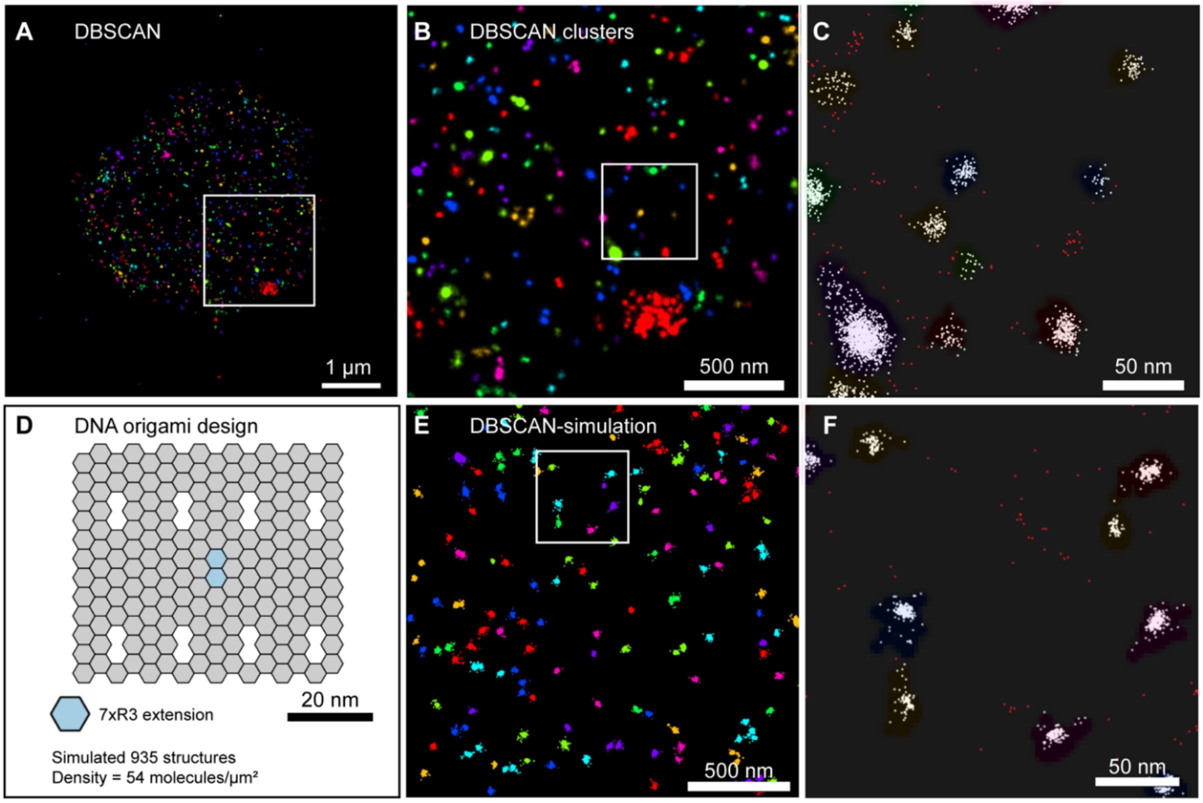

Supplementary Figure 4. DBSCAN shows some level of BCR clusters for the acquired data and monomers for the simulated data. (A) Representative result of clustering DNA-PAINT data from BCR imaging with DBSCAN. Different clusters are labeled in different colors. **(B)** Zoom-in of selected B cell clusters. **(C)** Overlay of DBSCAN cluster areas (light, colored) with the underlying DNA-PAINT data (red dots). **(D)** DNA origami design for BCR simulation. Two 7xR3 docking strands, representing a BCR molecule with two nanobodies bound, were placed at a 5 nm distance with Picasso Design. Random positions and rotations of these DNA origami structures were simulated at the same density as we detected for the BCRs on the B cell surface. **(E)** DBSCAN analysis of the simulated data shows mostly monomeric arrangements. **(F)** Zoom-in of selected simulated molecules. DBSCAN cluster areas (light, colored) are overlayed with the underlying DNA-PAINT data (red dots). The simulated data are representative of 3 independent simulations; the experimental data are representative of 3 independent experiments with n=31 cells. Source data are provided as Source Data file.

A

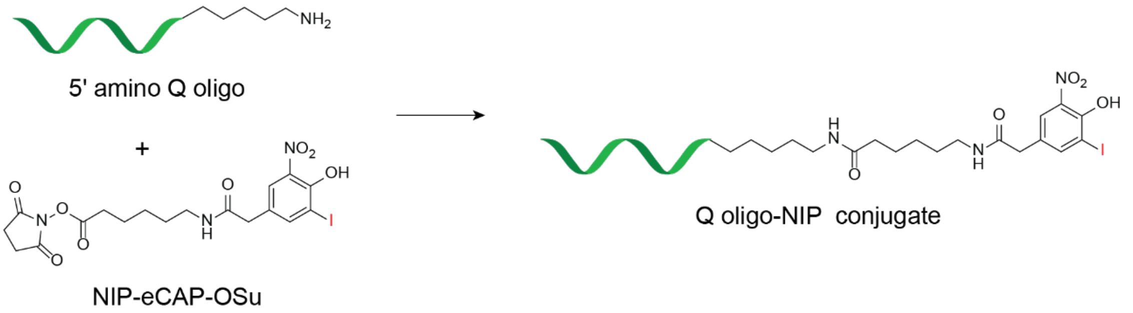

B

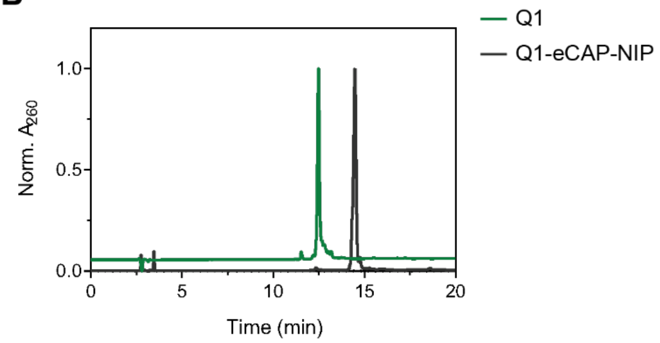

C

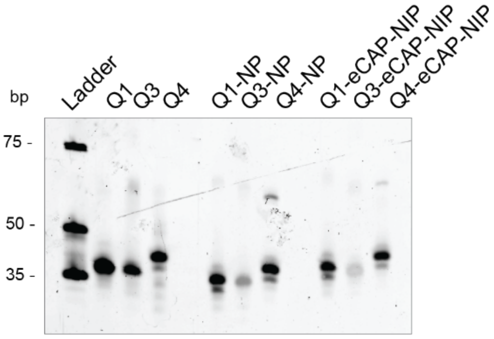

D

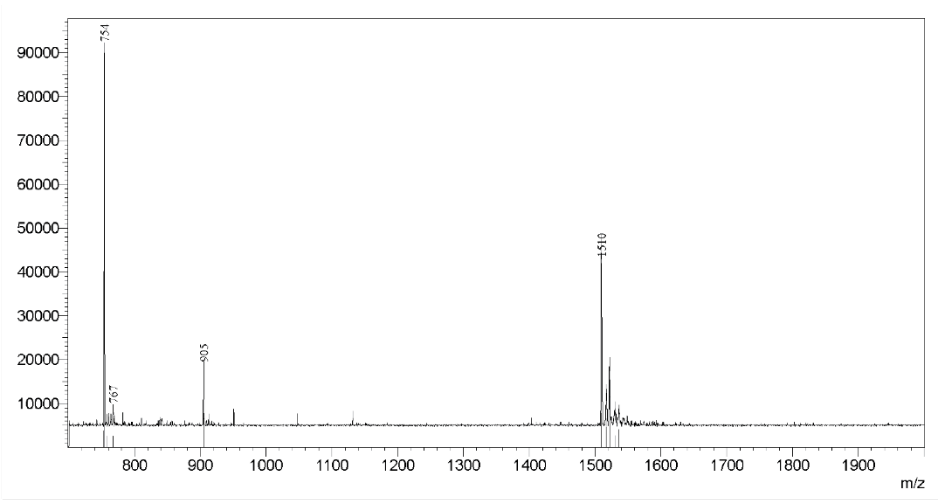

E

Conjugate	Predicted MW (g/mol)	Calculated MW (g/mol)
Q1-NP	4292.90	4291.95
Q3-NP	4316.93	4316.75
Q4-NP	4411.99	4410.66
Q1-eCAP-NIP	4531.96	4530.82
Q3-eCAP-NIP	4555.98	4554.45
Q4-eCAP-NIP	4651.04	4650.47

Supplementary Figure 5. Functionalization of Q oligos with NP or NIP haptens. (**A**) Reaction scheme showing the conjugation of NP hapten to the 5′ amino-modified Q oligo using an activated NHS ester. (**B**) A representative RP-HPLC chromatogram of Q1-eCAP-NIP conjugate purification. (**C**) A 12% denaturing PAGE gel indicating purified Q-NP and Q-eCAP-NIP (Q1-NIP) conjugates. Experiment was repeated independently two times with similar results. (**D**) Representative LC-MS mass chromatogram of purified Q1-eCAP-NIP conjugate. The purity of the sample is indicated by the low background and high signal-to-noise ratio. (**E**) Table showing predicted and calculated masses of oligo-hapten (NP or NIP) conjugates. The predicted masses were determined from the chemical structure of the conjugates drawn in the software ChemDraw Professional 17.1 (PerkinElmer). Calculated masses were derived from LC-MS analysis of purified conjugates using the analysis method H^+ with a tolerance of 1 Da. These data are in accordance with the predicted mass values. NIP-eCAP-OSu: 4-hydroxy-3-iodo-5-nitrophenylacetyl-O-succinimide ester; NP: 4-hydroxy-3-nitrophenylacetyl; NIP: 4-hydroxy-3-iodo-5-nitrophenylacetyl amino caproyl acetyl. Source data are provided as Source Data file.

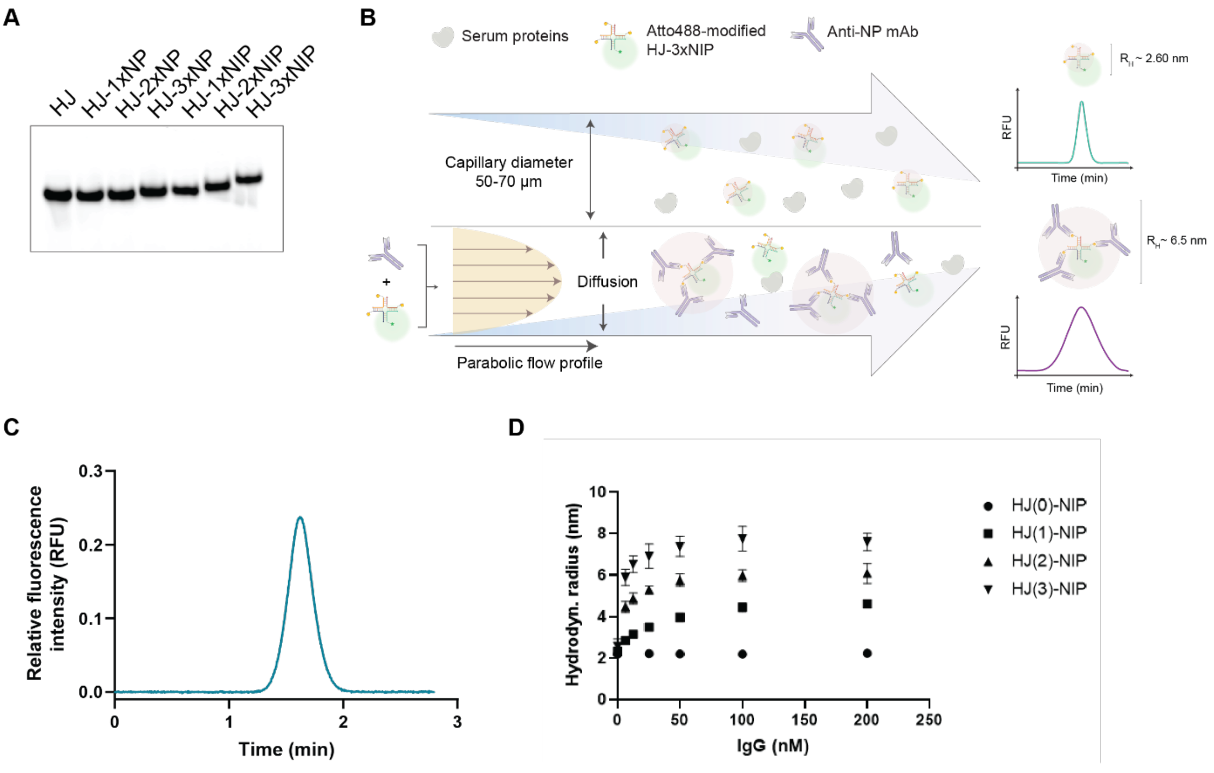

Supplementary Figure 6. Flow-induced dispersion analysis (FIDA) of HJ-NIP constructs. (**A**) A 12% native PAGE gel of Atto488-modified HJs displaying 0-3 units of NP or NIP used in FIDA analysis. As this is native PAGE, it was not possible to include a meaningful molecular weight marker. Experiment was repeated independently at least two times with similar results. (**B**) Schematic illustration of the principle of FIDA technology. In brief, a dye-labelled indicator (Atto488-modified HJ-3xNIP) alone or in combination with an analyte (anti-NP mAb) are injected into a capillary, where a parabolic flow profile of a buffer (typically PBS supplemented with BSA) is generated. In the capillary, the analyte is mixed with the indicator and as they diffuse toward the detector a Gaussian shaped signal, referred to as Taylorgram, appears. A complex formation between the analyte and indicator can be determined from the resultant signal compared to that of the indicator alone. As small molecules diffuse faster than larger ones, the shape of their signal will be more narrow than larger molecules or complexes. From the Taylorgrams, the apparent hydrodynamic radius (R_H) as well as dissociation constants (K_D) of individual molecules and complexes can be derived [33,34]. (**C**) A representative Taylorgram of Atto488-modfiied HJ-1xNP in Hanks Balanced Salt Solution (HBSS) supplemented with 0.1% BSA. (**D**) Apparent hydrodynamic radii of indicated AF488-labelled HJs (20 nM) as a function of an increasing anti-NP mAb concentration (0-200 nM) analyzed by FIDA in HBSS containing 0.1% BSA at pH 7.4. Each sample was pre-incubated for 30 min at RT. As seen, the apparent size of naked HJ remains the same at increasing anti-NP mAb

concentration, however HJs displaying 1-3 NIPs increase gradually in size corresponding to the number of mAbs each HJ-NIP construct can bind. For all three HJ-NIP variants, a plateau is reached at 100 nM anti-NP mAb, which indicates that no immune complex oligomerization (aggregate formation) is taking place. Thus, HJ-3xNIP can bind at most three mAbs, HJ-2xNIP two mAbs and HJ-1xNIP one mAb. Bars indicate mean ± SD of n=3 examined over at least three independent experiments. Source data are provided as Source Data file.

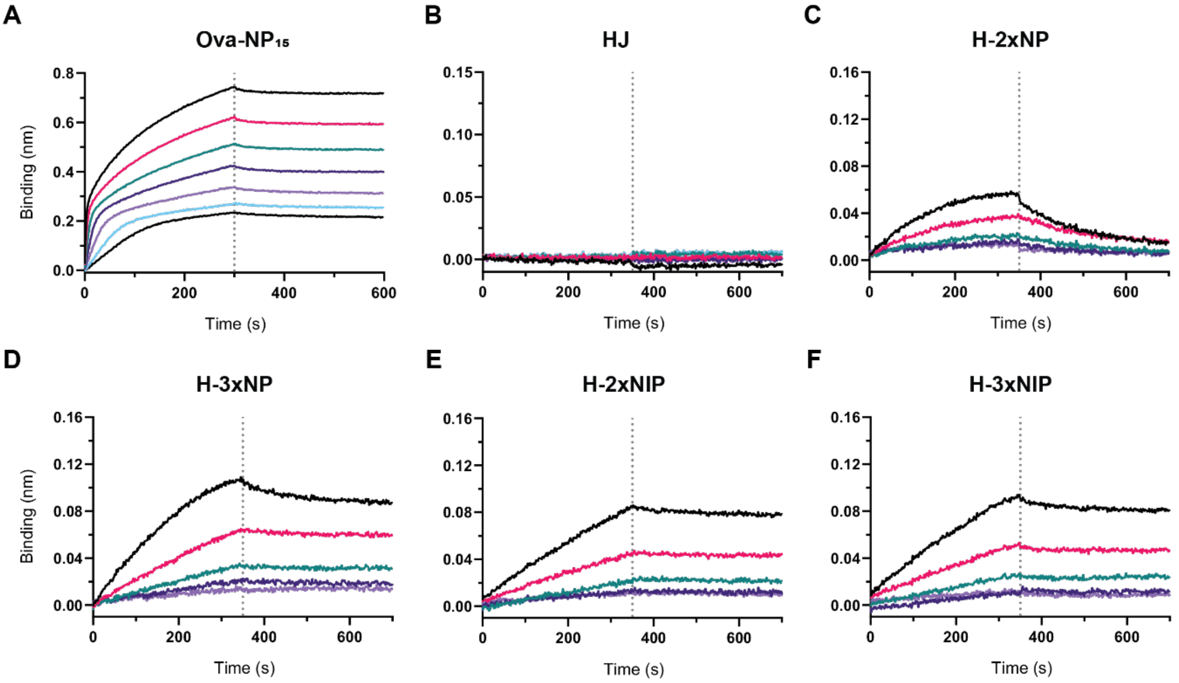

Supplementary Figure 7. Binding profile of haptenated HJs at low concentrations. (**A**) Ovalbumin modified with 15 NP molecules (Ova-NP_{15}) (3.25-240 nM) was used as a positive control for our initial bio-layer interferometry binding assays to confirm our setup and the anti-NP mAb immobilized via protein A biosensors. (**B**) Binding profile of naked HJ used as a negative control (0.313-20 nM). (**C-D**) Sensorgrams of HJ-2xNP or -3xNP in the low concentration range (0.125-2 nM). (**E-F**) Binding profiles of HJ-2xNIP and -3xNIP (0.063-1 nM). For all samples, the used concentration of anti-NP mAb, immobilized on the protein A sensors, was 1 µg/mL in a total volume of 200 µL. All samples were diluted in binding buffer in a 2-fold serial dilution in the described concentration intervals. Source data are provided as Source Data file.

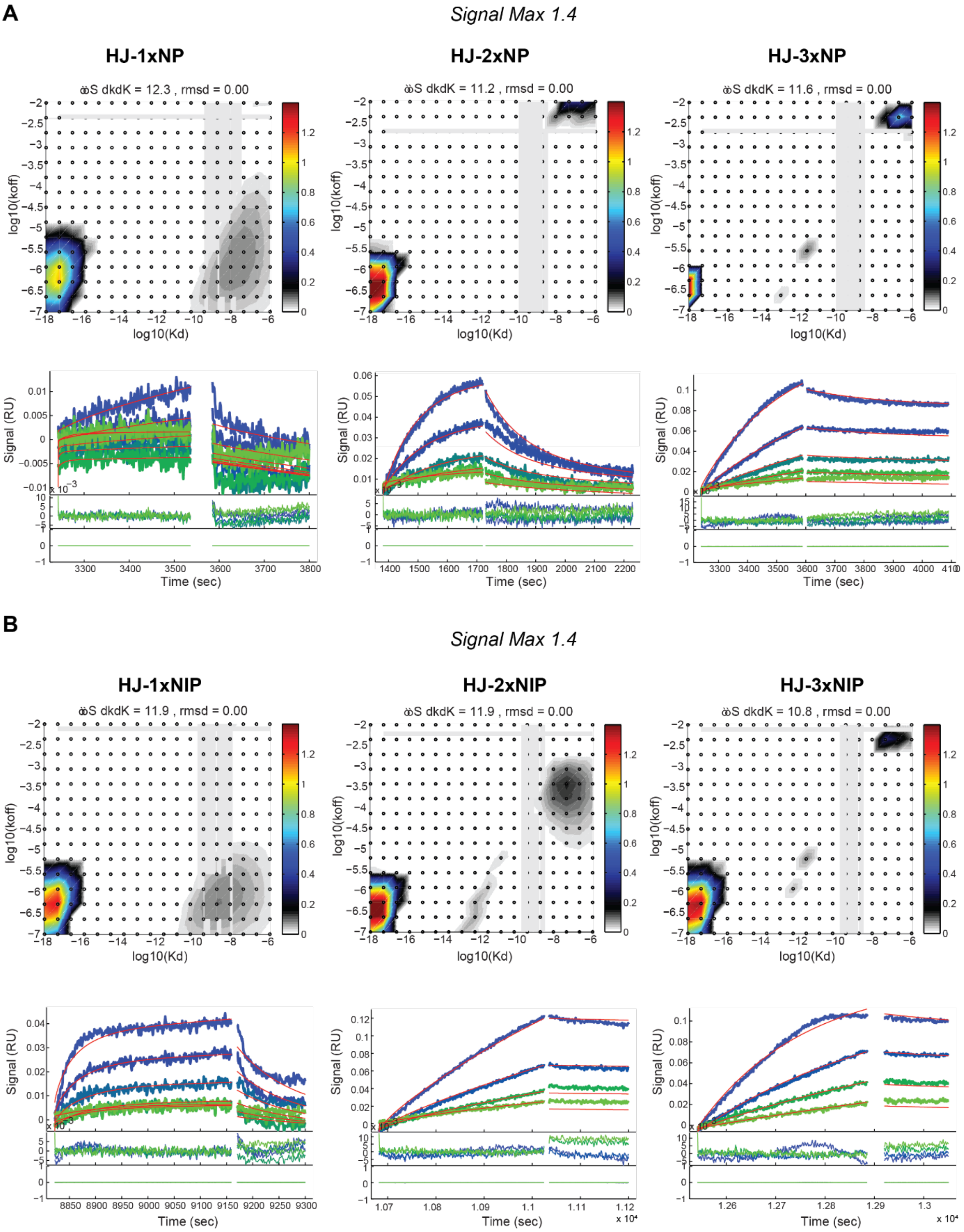

Supplementary Figure 8. EVILFIT analysis on HJ binding profile. (**A**) EVILFIT analysis of HJ-NP constructs based on following concentrations; HJ-1xNP (20, 10, 5, 2.5, 1.25, 0.625, 0.313 nM), HJ-2xNP and HJ-3xNP (5, 2.5, 1.25, 0.625, 0.313 nM). Upper panels show the unmagnified (signal max 1.4) 2D plots for individual HJ-NP. At this scale, only for HJ-3xNP

two small populations in the low K_D and slow off-rate (K_{off}) region are observed. Lower panels represent raw binding data and curve fits (red) that follow data points nicely as shown by residual plots below. (**B**) Upper panels indicate 2D EVILFIT plots based on BLI data using the following concentrations; HJ-1xNIP (7, 3.5, 1.75, 0.875, 0.438, 0.219 nM), HJ-2xNIP and HJ-3xNIP (1.75, 0.875, 0.438, 0.219 nM). Here, we see populations of low K_D and slow K_{off} for both HJ-2xNIP and -3xNIP. Lower panels show curve fitting of the raw data. For all samples, concentrations that resulted in the best possible fit were chosen. Source data are provided as Source Data file.

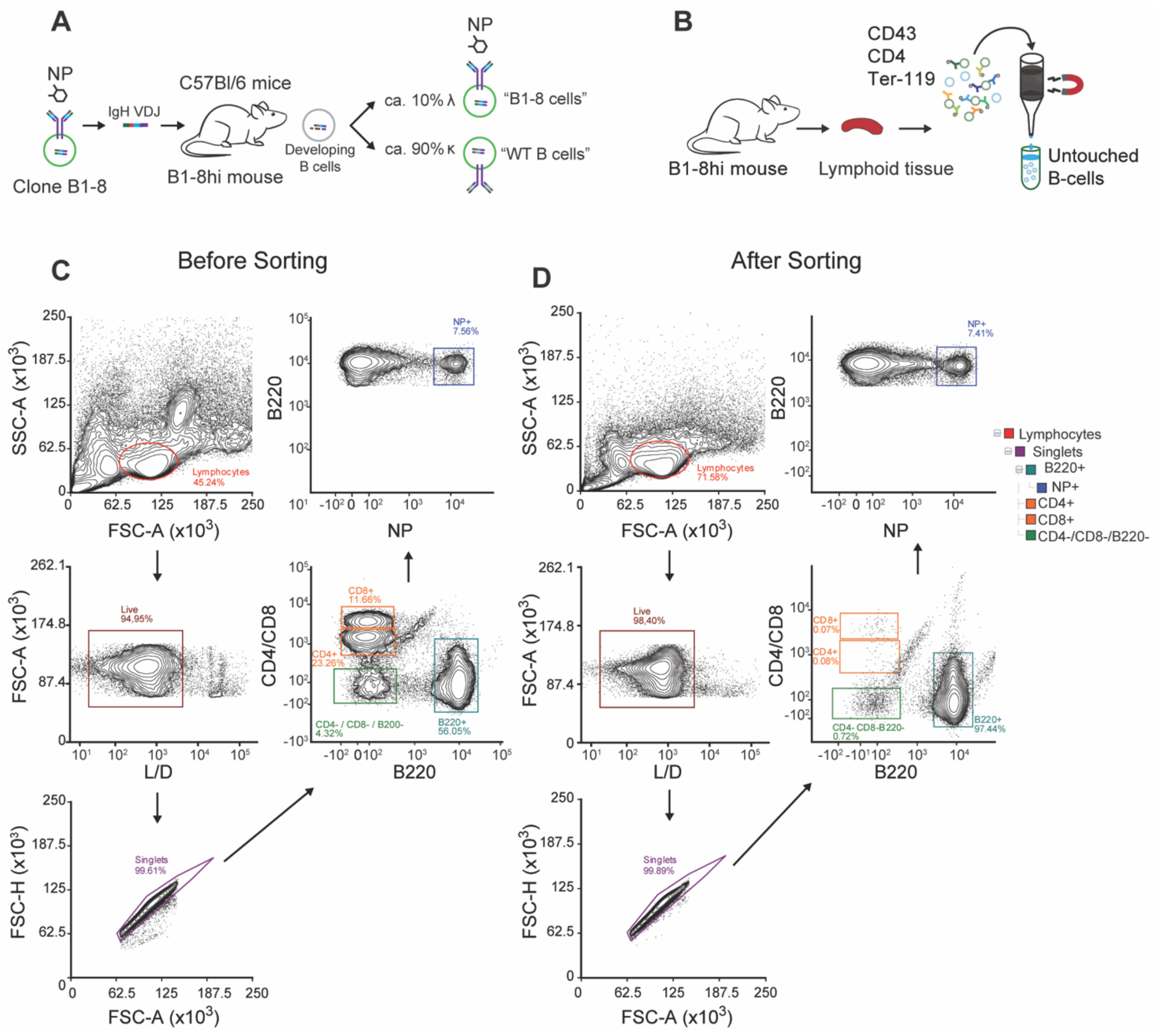

Supplementary Figure 9. Workflow for MACS purification of untouched B cells and subsequent purity analysis by flow cytometry. (**A**) B1-8hi mouse model. Heavy chain V(D)J fragment from a B1-8hi B cell clone was introduced into C57Bl/6 mice. The heavy chain of B cells developing in these mice will pair with either endogenous lambda (λ) light chain or kappa (κ) light chain. Only the ~12% of B cells expressing both the introduced heavy chain and λ light chain will be NP positive "B1-8hi B cells". (**B**) MACS purification of B1-8hi B cells. B cells were isolated using Magnetic-activated cell sorting (MACS). Lymphoid tissue from B1-8hi mice was processed using MACS sorting protocol. All cells except B cells were marked with magnetic beads using MACS sorting cocktail, resulting in untouched B cells flowing through the column unretained. (**C, D**) Flow cytometric purity analysis of MACS isolated cells. B1-8hi derived B cells were analysed for purity, using flow cytometry, after MACS separation. The frequency of B cells increased from 54% before sorting (panel C) to 97% after sorting

(panel D). Isolated B cells retained the subpopulation of essential B1-8hi NP positive cells (NP+). Data are representative of 3 independent experiments. Source data are provided as Source Data file.

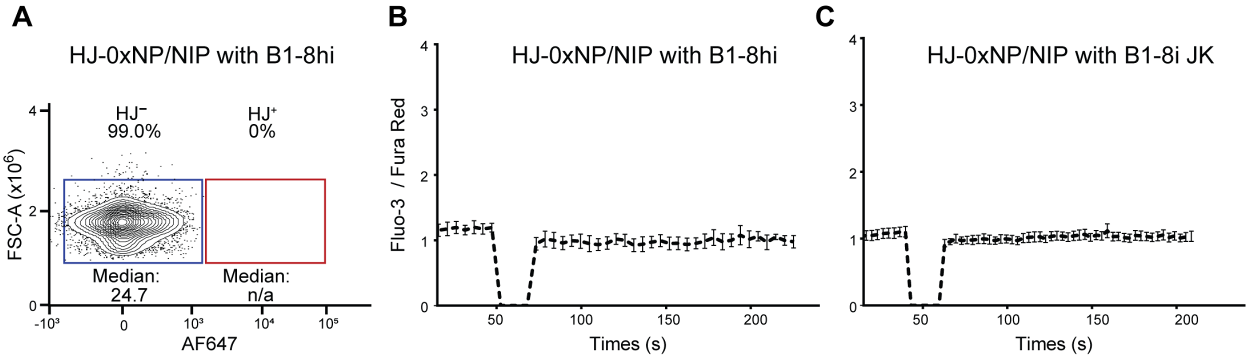

Supplementary Figure 10. Negative controls for flow cytometry and calcium flux. (**A**) Representative flow plot following incubation of B1-8hi cells with naked HJ (HJ-0xNP/NIP) at 2:1 ratio of HJ:BCR, showing absence of non-specific binding. (**B**) Calcium flux plot after addition of naked HJ (HJ-0xNP/NIP) to B1-8hi cells at 6000:1 ratio of HJ:BCR. (**C**) Calcium flux plot after addition of naked HJ (HJ-0xNP/NIP) to B1-8i cells at 1200:1 ratio of HJ:BCR. Data A are representative of 2 independent experiments and in B and C represent mean ± SD of 3 independent experiments. Source data are provided as Source Data file.

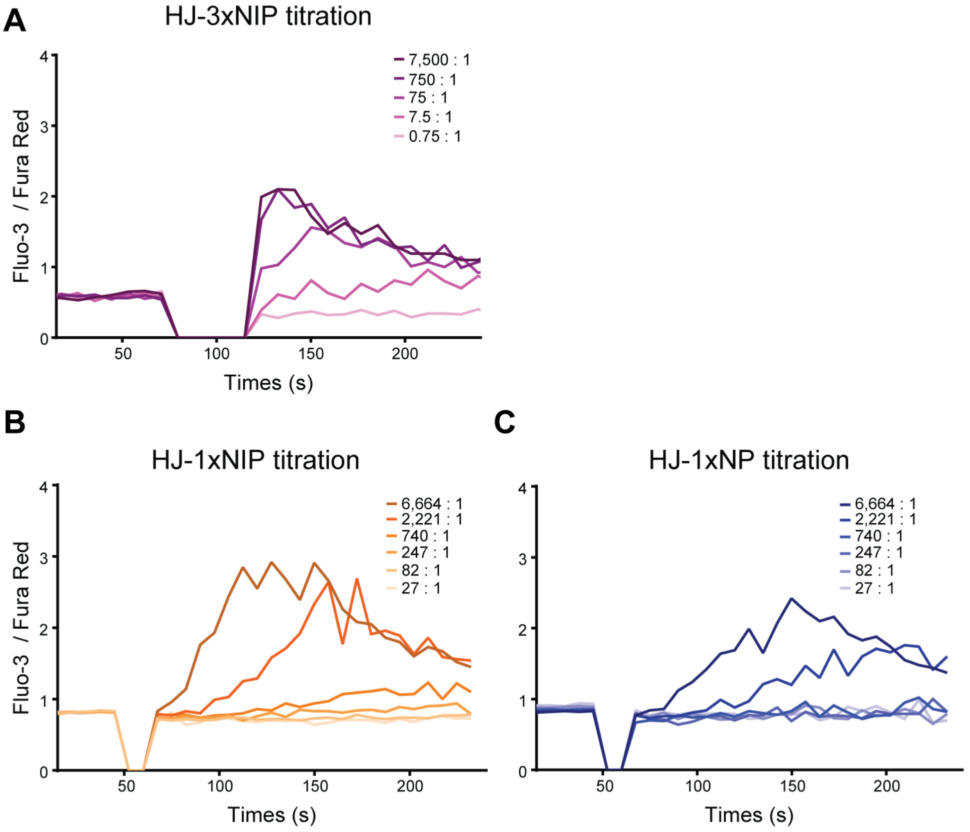

Supplementary Figure 11. Representative series of calcium flux traces following titration of HJs into wells containing B1-8hi B cells. (**A**) HJ-3xNIP titration. (**B**) HJ-1xNIP titration. (**C**) HJ-1xNP titration. Data are representative of 3 independent experiments. Source data are provided as Source Data file.

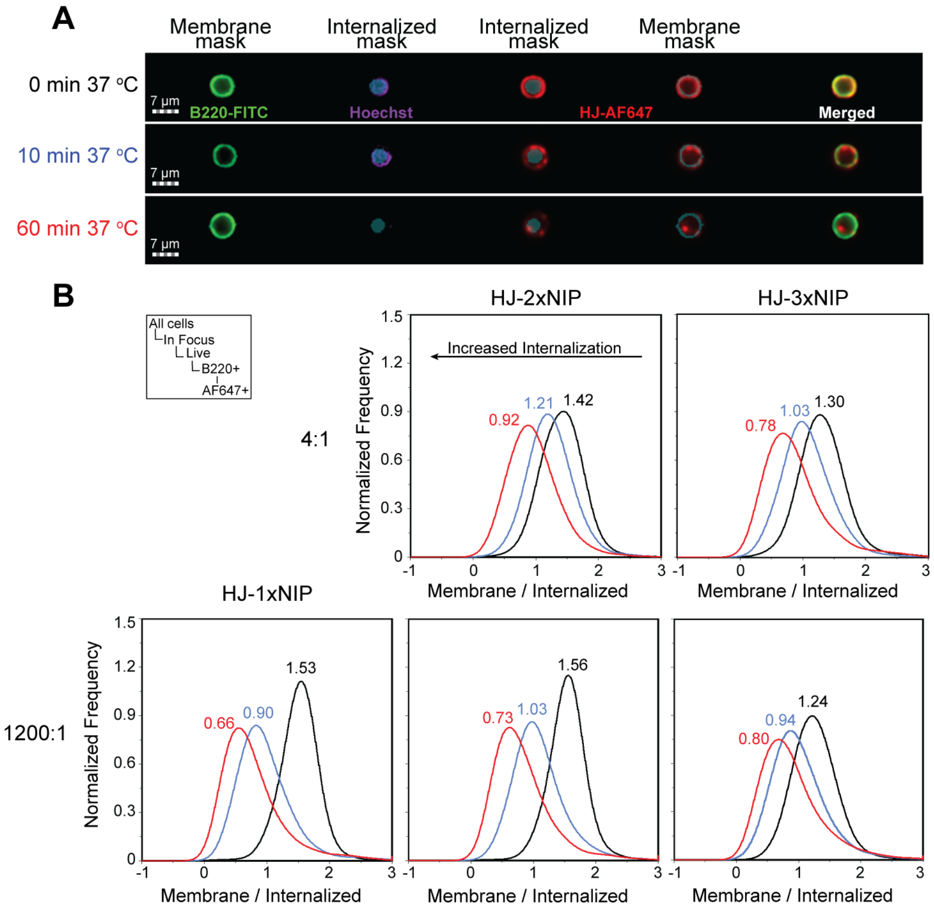

Supplementary Figure 12. Uptake and internalization of HJs by B1-8hi B cells measured by imaging flow cytometry. (**A**) As also shown in main Figure 5, a masking method was used to measure the amount of HJ signal (red) on the surface of the cells and within the cells. Membrane and internalization masks were made based on the B220 marker (green), either overlapping with it or excluding it. Nuclear Hoechst stain (blue) shows the potential overlap of internalized mask with the nucleus. (**B**) AF647 signal from different HJ constructs within these masks was quantified and plotted as membrane/internalized ratio for different valencies, stoichiometries, as well as incubation conditions (time at 37 °C) (no signal was detected for HJ-1xNIP at the low stoichiometry). The curves represent between 3,000 and 5,000 individual cells, and the median is given above each curve. Data are shown from one out of three independent experiments. Source data are provided as Source Data file.

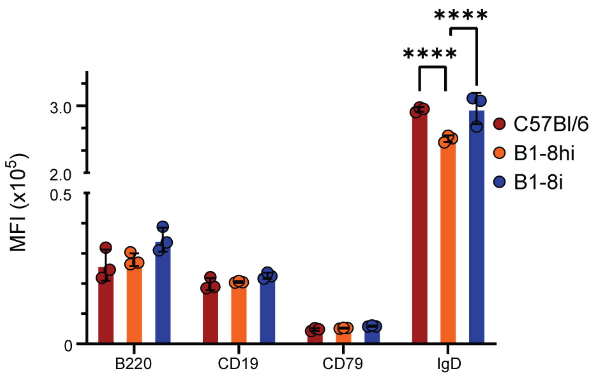

Supplementary Figure 13. Flow cytometric evaluation of surface expression of BCR constituents and associated molecules in C57Bl/6, B1-8hi and B1-8i models. Median fluorescence intensities for B220, CD19, CD79 and IgD were evaluated upon flow cytometric analysis of splenocytes from C57Bl/6, B1-8hi and B1-8i models. Mean ± SD from 3 individual mice per group. Statistical analysis was performed using two-way ANOVA with Tukey's post-test using alpha = 0.05; **** = p<0.0001. Source data are provided as Source Data file.

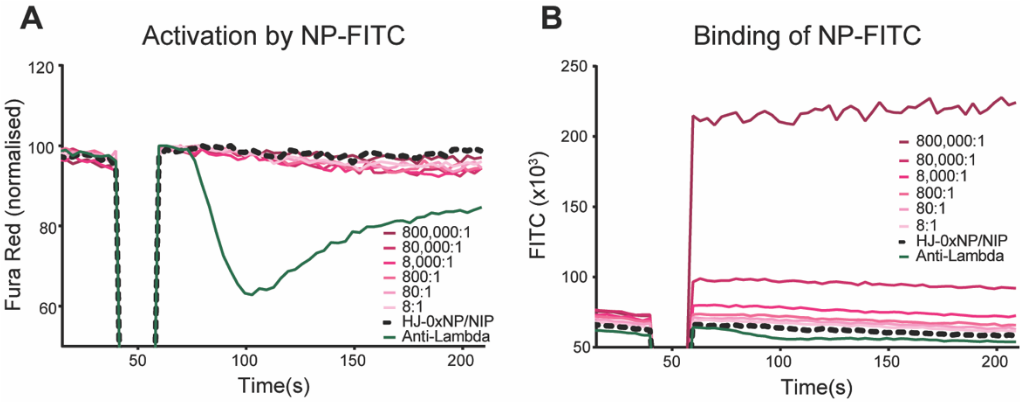

Supplementary Figure 14. Calcium flux traces and binding curves for NP-FITC and controls upon incubation with B1-8i cells. (**A**) Calcium flux traces following titration of NP-FITC into wells containing B1-8i cells. An anti-lambda chain antibody was used as positive control (decrease in Fura Red signal), and naked HJ as negative control (no change from baseline). (**B**) FITC signal for binding of NP-FITC concomitant with the read-out for calcium flux. Anti-lambda chain antibody and naked HJ both serve as negative controls. Data are shown from one out of two independent experiments. Source data are provided as Source Data file.

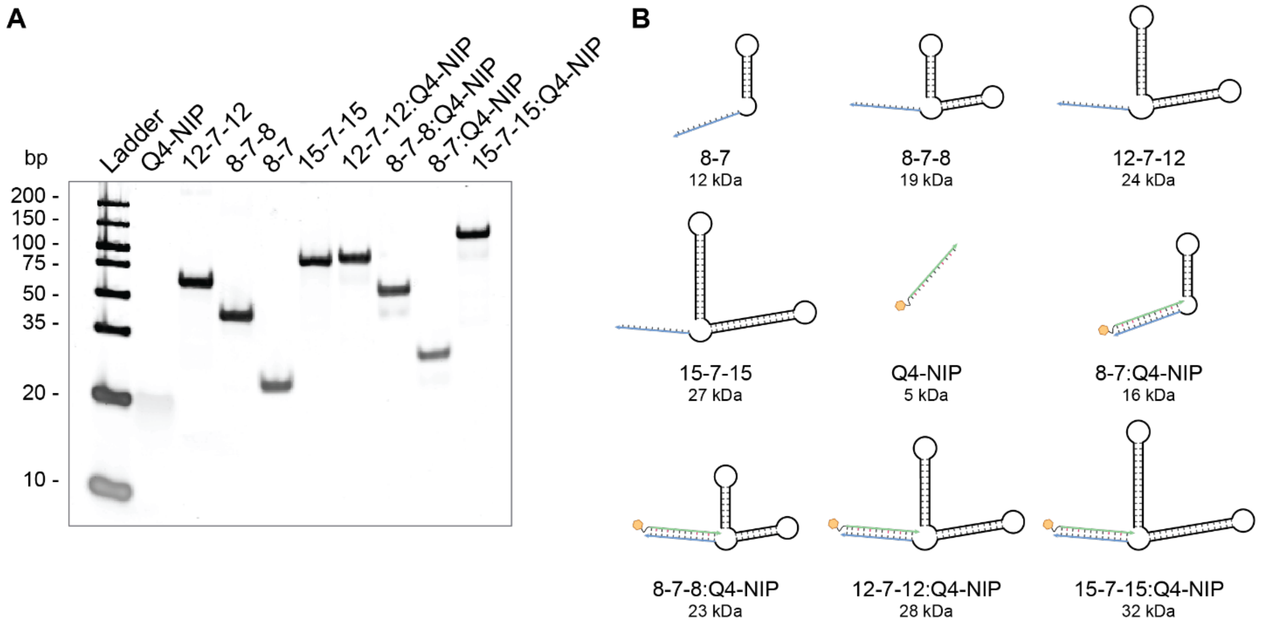

Supplementary Figure 15. Generation and assembly of antigenic dumbbell structures of varying sizes. (**A**) Gel showing the migration of the 4 dumbbell structures alone or after assembly with Q4-1NIP. Experiment was repeated independently two times with similar results. (**B**) Schematic 2D representation of the 4 dumbbells, Q4-NIP and the 4 dumbbells assembled with Q4-NIP. Source data are provided as Source Data file.

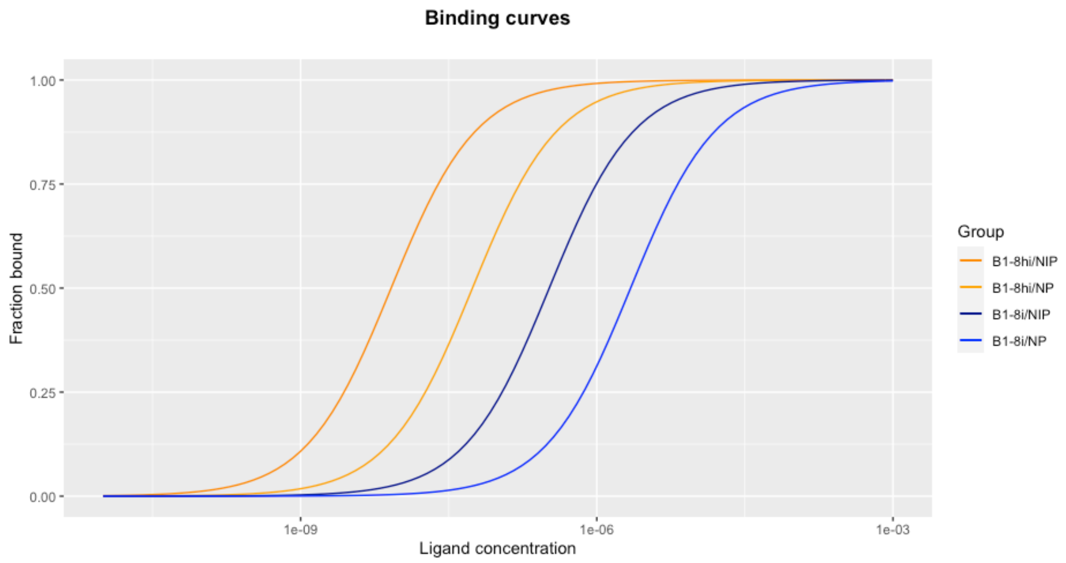

Supplementary Figure 16. Calculated binding curves for B1-8hi/NIP, B1-8hi/NP, B1-8i/NIP, and B1-8i/NP. Binding curves were based on *eq2*, with x as the total concentration of antigen, a equal to the constant 8.30×10^{-11}, which is modified by y, representing the fraction of cells carrying receptors specific for the antigen, and finally z is the K_D of the antigen-receptor pair being interrogated. This was plotted in R across 8 orders of magnitude from 10^{-11} to 10^{-3} for B1-8hi/NIP (freqB1-8=0.12, K_D=8.25×10^{-9}), B1-8hi/NP (freqB1-8=0.12, K_D=5.5×10^{-8}), B1-8i/NIP (freqB1-8=0.5, K_D=3.3×10^{-7}), and B1-i/NP (freqB1-8=0.5, K_D=2.2×10^{-6}). Curves were generated in R using the indicated parameters and the script provided in the Dryad data deposit associated with the article.

Name	Sequence (5′ - 3′)	5′-mod	3′- mod
**7xR3 docking strand**	CTCTCTCTCTCTCTCTCTC	Azide	
**Extension on DNA origami S1-hybridization (S1HP3H)**	Staple-TTGTGATGTAGGTGGTAGAGGAA	Staple	
**Antibody-conjugation Strand with P3 handle (DBCO-S1-P3)**	TTCCTCTACCACCTACATCACTTTCTTCATTA	DBCO	
**Control strand (S1-P3 handle)**	ATACATCTATTTTCCTCTACCACCTACAT CACTTTCTTCATTA		
**R3* imager strand**	GAG AGAG		Cy3b
**P3* imager strand**	GTAATGAAGA		Cy3b

Supplementary Table 1. Reagents used for DNA-PAINT imaging. The table provides an overview of the DNA sequences with different chemical handles used for coupling to the κLC-Nb and the mIgG2a for DNA-PAINT imaging.

Name	Sequence (5′ - 3′)
21[32]23[31]BLK	TTTTCACTCAAAGGGCGAAAAACCATCACC
19[32]21[31]BLK	GTCGACTTCGGCCAACGCGCGGGGTTTTTC
17[32]19[31]BLK	TGCATCTTTCCCAGTCACGACGGCCTGCAG
15[32]17[31]BLK	TAATCAGCGGATTGACCGTAATCGTAACCG
13[32]15[31]BLK	AACGCAAAATCGATGAACGGTACCGGTTGA
11[32]13[31]BLK	AACAGTTTTGTACCAAAAACATTTTATTTC
9[32]11[31]BLK	TTTACCCCAACATGTTTTAAATTTCCATAT
7[32]9[31]BLK	TTTAGGACAAATGCTTTAAACAATCAGGTC
5[32]7[31]BLK	CATCAAGTAAAACGAACTAACGAGTTGAGA
3[32]5[31]BLK	AATACGTTTGAAAGAGGACAGACTGACCTT
1[32]3[31]BLK	AGGCTCCAGAGGCTTTGAGGACACGGGTAA
0[47]1[31]BLK	AGAAAGGAACAACTAAAGGAATTCAAAAAAA
23[32]22[48]BLK	CAAATCAAGTTTTTTGGGGTCGAAACGTGGA
22[47]20[48]BLK	CTCCAACGCAGTGAGACGGGCAACCAGCTGCA
20[47]18[48]BLK	TTAATGAACTAGAGGATCCCCGGGGGGTAACG
18[47]16[48]BLK	CCAGGGTTGCCAGTTTGAGGGGACCCGTGGGA
16[47]14[48]BLK	ACAAACGGAAAAGCCCCAAAAACACTGGAGCA
14[47]12[48]BLK	AACAAGAGGGATAAAAATTTTTAGCATAAAGC
12[47]10[48]BLK	TAAATCGGGATTCCCAATTCTGCGATATAATG
10[47]8[48]BLK	CTGTAGCTTGACTATTATAGTCAGTTCATTGA
8[47]6[48]BLK	ATCCCCCTATACCACATTCAACTAGAAAAATC
6[47]4[48]BLK	TACGTTAAAGTAATCTTGACAAGAACCGAACT
4[47]2[48]BLK	GACCAACTAATGCCACTACGAAGGGGGTAGCA
2[47]0[48]BLK	ACGGCTACAAAAGGAGCCTTTAATGTGAGAAT
21[56]23[63]BLK	AGCTGATTGCCCTTCAGAGTCCACTATTAAAGGGTGCCGT
15[64]18[64]BLK	GTATAAGCCAACCCGTCGGATTCTGACGACAGTATCGGCCGCAAGGCG
13[64]15[63]BLK	TATATTTTGTCATTGCCTGAGAGTGGAAGATT
11[64]13[63]BLK	GATTTAGTCAATAAAGCCTCAGAGAACCCTCA
9[64]11[63]BLK	CGGATTGCAGAGCTTAATTGCTGAAACGAGTA
7[56]9[63]BLK	ATGCAGATACATAACGGGAATCGTCATAAATAAAGCAAAG
1[64]4[64]BLK	TTTATCAGGACAGCATCGGAACGACACCAACCTAAAACGAGGTCAATC
0[79]1[63]BLK	ACAACTTTCAACAGTTTCAGCGGATGTATCGG
23[64]22[80]BLK	AAAGCACTAAATCGGAACCCTAATCCAGTT

22[79]20[80]BLK	TGGAACAACCGCCTGGCCCTGAGGCCCGCT
20[79]18[80]BLK	TTCCAGTCGTAATCATGGTCATAAAAGGGG
18[79]16[80]BLK	GATGTGCTTCAGGAAGATCGCACAATGTGA
16[79]14[80]BLK	GCGAGTAAAAATATTTAAATTGTTACAAAG
14[79]12[80]BLK	GCTATCAGAAATGCAATGCCTGAATTAGCA
12[79]10[80]BLK	AAATTAAGTTGACCATTAGATACTTTTGCG
10[79]8[80]BLK	GATGGCTTATCAAAAAGATTAAGAGCGTCC
8[79]6[80]BLK	AATACTGCCCAAAAGGAATTACGTGGCTCA
6[79]4[80]BLK	TTATACCACCAAATCAACGTAACGAACGAG
4[79]2[80]BLK	GCGCAGACAAGAGGCAAAAGAATCCCTCAG
2[79]0[80]BLK	CAGCGAAACTTGCTTTCGAGGTGTTGCTAA
21[96]23[95]BLK	AGCAAGCGTAGGGTTGAGTGTTGTAGGGAGCC
19[96]21[95]BLK	CTGTGTGATTGCGTTGCGCTCACTAGAGTTGC
17[96]19[95]BLK	GCTTTCCGATTACGCCAGCTGGCGGCTGTTTC
15[96]17[95]BLK	ATATTTTGGCTTTCATCAACATTATCCAGCCA
13[96]15[95]BLK	TAGGTAAACTATTTTTGAGAGATCAAACGTTA
11[96]13[95]BLK	AATGGTCAACAGGCAAGGCAAAGAGTAATGTG
9[96]11[95]BLK	CGAAAGACTTTGATAAGAGGTCATATTTCGCA
7[96]9[95]BLK	TAAGAGCAAATGTTTAGACTGGATAGGAAGCC
5[96]7[95]BLK	TCATTCAGATGCGATTTTAAGAACAGGCATAG
3[96]5[95]BLK	ACACTCATCCATGTTACTTAGCCGAAAGCTGC
1[96]3[95]BLK	AAACAGCTTTTTGCGGGATCGTCAACACTAAA
0[111]1[95]BLK	TAAATGAATTTTCTGTATGGGATTAATTTCTT
23[96]22[112]BLK	CCCGATTTAGAGCTTGACGGGGAAAAAGAATA
22[111]20[112]BLK	GCCCGAGAGTCCACGCTGGTTTGCAGCTAACT
20[111]18[112]BLK	CACATTAAAATTGTTATCCGCTCATGCGGGCC
18[111]16[112]BLK	TCTTCGCTGCACCGCTTCTGGTGCGGCCTTCC
16[111]14[112]BLK	TGTAGCCATTAAAATTCGCATTAAATGCCGGA
14[111]12[112]BLK	GAGGGTAGGATTCAAAAGGGTGAGACATCCAA
12[111]10[112]BLK	TAAATCATATAACCTGTTTAGCTAACCTTTAA
10[111]8[112]BLK	TTGCTCCTTTCAAATATCGCGTTTGAGGGGGT
8[111]6[112]BLK	AATAGTAAACACTATCATAACCCTCATTGTGA
6[111]4[112]BLK	ATTACCTTTGAATAAGGCTTGCCCAAATCCGC
4[111]2[112]BLK	GACCTGCTCTTTGACCCCCAGCGAGGGAGTTA
2[111]0[112]BLK	AAGGCCGCTGATACCGATAGTTGCGACGTTAG
21[120]23[127]BLK	CCCAGCAGGCGAAAAATCCCTTATAAATCAAGCCGGCG
15[128]18[128]BLK	TAAATCAAAATAATTCGCGTCTCGGAAACCAGGCAAAGGGAAGG
13[128]15[127]BLK	GAGACAGCTAGCTGATAAATTAATTTTTGT
11[128]13[127]BLK	TTTGGGGATAGTAGTAGCATTAAAAGGCCG

9[128]11[127]BLK	GCTTCAATCAGGATTAGAGAGTTATTTTCA
7[120]9[127]BLK	CGTTTACCAGACGACAAAGAAGTTTTGCCATAATTCGA
1[128]4[128]BLK	TGACAACTCGCTGAGGCTTGCATTATACCAAGCGCGATGATAAA
0[143]1[127]BLK	TCTAAAGTTTTGTCGTCTTTCCAGCCGACAA
21[160]22[144]BLK	TCAATATCGAACCTCAAATATCAATTCCGAAA
19[160]20[144]BLK	GCAATTCACATATTCCTGATTATCAAAGTGTA
17[160]18[144]BLK	AGAAAACAAAGAAGATGATGAAACAGGCTGCG
15[160]16[144]BLK	ATCGCAAGTATGTAAATGCTGATGATAGGAAC
13[160]14[144]BLK	GTAATAAGTTAGGCAGAGGCATTTATGATATT
11[160]12[144]BLK	CCAATAGCTCATCGTAGGAATCATGGCATCAA
9[160]10[144]BLK	AGAGAGAAAAAAATGAAAATAGCAAGCAAACT
7[160]8[144]BLK	TTATTACGAAGAACTGGCATGATTGCGAGAGG
5[160]6[144]BLK	GCAAGGCCTCACCAGTAGCACCATGGGCTTGA
3[160]4[144]BLK	TTGACAGGCCACCACCAGAGCCGCGATTTGTA
1[160]2[144]BLK	TTAGGATTGGCTGAGACTCCTCAATAACCGAT
0[175]0[144]BLK	TCCACAGACAGCCCTCATAGTTAGCGTAACGA
23[128]23[159]BLK	AACGTGGCGAGAAAGGAAGGGAAACCAGTAA
22[143]21[159]BLK	TCGGCAAATCCTGTTTGATGGTGGACCCTCAA
20[143]19[159]BLK	AAGCCTGGTACGAGCCGGAAGCATAGATGATG
18[143]17[159]BLK	CAACTGTTGCGCCATTCGCCATTCAAACATCA
16[143]15[159]BLK	GCCATCAAGCTCATTTTTTAACCACAAATCCA
14[143]13[159]BLK	CAACCGTTTCAAATCACCATCAATTCGAGCCA
12[143]11[159]BLK	TTCTACTACGCGAGCTGAAAAGGTTACCGCGC
10[143]9[159]BLK	CCAACAGGAGCGAACCAGACCGGAGCCTTTAC
8[143]7[159]BLK	CTTTTGCAGATAAAAACCAAAATAAAGACTCC
6[143]5[159]BLK	GATGGTTTGAACGAGTAGTAAATTTACCATTA
4[143]3[159]BLK	TCATCGCCAACAAAGTACAACGGACGCCAGCA
2[143]1[159]BLK	ATATTCGGAACCATCGCCCACGCAGAGAAGGA
23[160]22[176]BLK	TAAAAGGGACATTCTGGCCAACAAAGCATC
22[175]20[176]BLK	ACCTTGCTTGGTCAGTTGGCAAAGAGCGGA
20[175]18[176]BLK	ATTATCATTCAATATAATCCTGACAATTAC
18[175]16[176]BLK	CTGAGCAAAAATTAATTACATTTTGGGTTA
16[175]14[176]BLK	TATAACTAACAAAGAACGCGAGAACGCCAA
14[175]12[176]BLK	CATGTAATAGAATATAAAGTACCAAGCCGT
12[175]10[176]BLK	TTTTATTTAAGCAAATCAGATATTTTTTGT
10[175]8[176]BLK	TTAACGTCTAACATAAAAACAGGTAACGGA
8[175]6[176]BLK	ATACCCAACAGTATGTTAGCAAATTAGAGC
6[175]4[176]BLK	CAGCAAAAGGAAACGTCACCAATGAGCCGC
4[175]2[176]BLK	CACCAGAAAGGTTGAGGCAGGTCATGAAAG

2[175]0[176]BLK	TATTAAGAAGCGGGGTTTTGCTCGTAGCAT
21[184]23[191]BLK	TCAACAGTTGAAAGGAGCAAATGAAAAATCTAGAGATAGA
15[192]18[192]BLK	TCAAATATAACCTCCGGCTTAGGTAACAATTTCATTTGAAGGCGAATT
13[192]15[191]BLK	GTAAAGTAATCGCCATATTTAACAAAACTTTT
11[192]13[191]BLK	TATCCGGTCTCATCGAGAACAAGCGACAAAAG
9[192]11[191]BLK	TTAGACGGCCAAATAAGAAACGATAGAAGGCT
7[184]9[191]BLK	CGTAGAAAATACATACCGAGGAAACGCAATAAGAAGCGCA
1[192]4[192]BLK	GCGGATAACCTATTATTCTGAAACAGACGATTGGCCTTGAAGAGCCAC
0[207]1[191]BLK	TCACCAGTACAAACTACAACGCCTAGTACCAG
23[192]22[208]BLK	ACCCTTCTGACCTGAAAGCGTAAGACGCTGAG
22[207]20[208]BLK	AGCCAGCAATTGAGGAAGGTTATCATCATTTT
20[207]18[208]BLK	GCGGAACATCTGAATAATGGAAGGTACAAAAT
18[207]16[208]BLK	CGCGCAGATTACCTTTTTTAATGGGAGAGACT
16[207]14[208]BLK	ACCTTTTTATTTTAGTTAATTTCATAGGGCTT
14[207]12[208]BLK	AATTGAGAATTCTGTCCAGACGACTAAACCAA
12[207]10[208]BLK	GTACCGCAATTCTAAGAACGCGAGTATTATTT
10[207]8[208]BLK	ATCCCAATGAGAATTAACTGAACAGTTACCAG
8[207]6[208]BLK	AAGGAAACATAAAGGTGGCAACATTATCACCG
6[207]4[208]BLK	TCACCGACGCACCGTAATCAGTAGCAGAACCG
4[207]2[208]BLK	CCACCCTCTATTCACAAACAAATACCTGCCTA
2[207]0[208]BLK	TTTCGGAAGTGCCGTCGAGAGGGTGAGTTTCG
21[224]23[223]BLK	CTTTAGGGCCTGCAACAGTGCCAATACGTG
19[224]21[223]BLK	CTACCATAGTTTGAGTAACATTTAAAATAT
17[224]19[223]BLK	CATAAATCTTTGAATACCAAGTGTTAGAAC
15[224]17[223]BLK	CCTAAATCAAAATCATAGGTCTAAACAGTA
13[224]15[223]BLK	ACAACATGCCAACGCTCAACAGTCTTCTGA
11[224]13[223]BLK	GCGAACCTCCAAGAACGGGTATGACAATAA
9[224]11[223]BLK	AAAGTCACAAAATAAACAGCCAGCGTTTTA
7[224]9[223]BLK	AACGCAAAGATAGCCGAACAAACCCTGAAC
5[224]7[223]BLK	TCAAGTTTCATTAAAGGTGAATATAAAAGA
3[224]5[223]BLK	TTAAAGCCAGAGCCGCCACCCTCGACAGAA
1[224]3[223]BLK	GTATAGCAAACAGTTAATGCCCAATCCTCA
0[239]1[223]BLK	AGGAACCCATGTACCGTAACACTTGATATAA
23[224]22[240]BLK	GCACAGACAATATTTTTGAATGGGGTCAGTA
22[239]20[240]BLK	TTAACACCAGCACTAACAACTAATCGTTATTA
20[239]18[240]BLK	ATTTTAAAATCAAAATTATTTGCACGGATTCG
18[239]16[240]BLK	CCTGATTGCAATATATGTGAGTGATCAATAGT

16[239]14[240]BLK	GAATTTATTTAATGGTTTGAAATATTCTTACC
14[239]12[240]BLK	AGTATAAAGTTCAGCTAATGCAGATGTCTTTC
12[239]10[240]BLK	CTTATCATTCCCGACTTGCGGGAGCCTAATTT
10[239]8[240]BLK	GCCAGTTAGAGGGTAATTGAGCGCTTTAAGAA
8[239]6[240]BLK	AAGTAAGCAGACACCACGGAATAATATTGACG
6[239]4[240]BLK	GAAATTATTGCCTTTAGCGTCAGACCGGAACC
4[239]2[240]BLK	GCCTCCCTCAGAATGGAAAGCGCAGTAACAGT
2[239]0[240]BLK	GCCCGTATCCGGAATAGGTGTATCAGCCCAAT
21[248]23[255]BLK	AGATTAGAGCCGTCAAAAAACAGAGGTGAGGCCTATTAGT
15[256]18[256]BLK	GTGATAAAAAGACGCTGAGAAGAGATAACCTTGCTTCTGTTCGGGAGA
13[256]15[255]BLK	GTTTATCAATATGCGTTATACAAACCGACCGT
11[256]13[255]BLK	GCCTTAAACCAATCAATAATCGGCACGCGCCT
9[256]11[255]BLK	GAGAGATAGAGCGTCTTTCCAGAGGTTTTGAA
7[248]9[255]BLK	GTTTATTTTGTCACAATCTTACCGAAGCCCTTTAATATCA
1[256]4[256]BLK	CAGGAGGTGGGGTCAGTGCCTTGAGTCTCTGAATTTACCGGGAACCAG
0[271]1[255]BLK	CCACCCTCATTTTCAGGGATAGCAACCGTACT
23[256]22[272]BLK	CTTTAATGCGCGAACTGATAGCCCCACCAG
22[271]20[272]BLK	CAGAAGATTAGATAATACATTTGTCGACAA
20[271]18[272]BLK	CTCGTATTAGAAATTGCGTAGATACAGTAC
18[271]16[272]BLK	CTTTTACAAAATCGTCGCTATTAGCGATAG
16[271]14[272]BLK	CTTAGATTTAAGGCGTTAAATAAAGCCTGT
14[271]12[272]BLK	TTAGTATCACAATAGATAAGTCCACGAGCA
12[271]10[272]BLK	TGTAGAAATCAAGATTAGTTGCTCTTACCA
10[271]8[272]BLK	ACGCTAACACCCACAAGAATTGAAAATAGC
8[271]6[272]BLK	AATAGCTATCAATAGAAAATTCAACATTCA
6[271]4[272]BLK	ACCGATTGTCGGCATTTTCGGTCATAATCA
4[271]2[272]BLK	AAATCACCTTCCAGTAAGCGTCAGTAATAA
2[271]0[272]BLK	GTTTTAACTTAGTACCGCCACCCAGAGCCA

Supplementary Table 2. DNA strands for DNA origami folding. List of staple strands and their sequences used for a DNA origami with the 40 nm structure. The sequences highlighted in green indicate the staple strands used for immobilization of the four antibodies for determination of the nanobody labelling efficiency.

	mean	RMSE			
		B1-8hi:NIP	B1-8hi:NP	B1-8i:NIP	B1-8i:NP
B1-8hi:NIP	0.657	-	0.175	0.320	0.444
B1-8hi:NP	0.554	-	-	0.166	0.319
B1-8i:NIP	0.457	-	-	-	0.176
B1-8i:NP	0.354	-	-	-	-

Supplementary Table 3. Example data for binding curve simulations using one randomized vector. We generated a vector of 80,000 values, distributed with 10,000 random values across each of the 8 logarithmic intervals from 10^{-11} to 10^{-3}. We then calculated the fraction bound for the numbers in this vector using each of the binding curves given in Fig. S16, then calculated the mean, and finally the root mean square error (RMSE).

	Mean	RMSE			
		+10%	-10%	+20%	-20%
B1-8hi:NIP	0.657	6.35×10^{-6}	6.35×10^{-6}	1.27×10^{-5}	1.27×10^{-5}
B1-8hi:NP	0.554	9.35×10^{-7}	9.35×10^{-7}	1.87×10^{-6}	1.87×10^{-6}
B1-8i:NIP	0.457	6.44×10^{-7}	6.44×10^{-7}	1.29×10^{-6}	1.29×10^{-6}
B1-8i:NP	0.354	9.77×10^{-8}	9.77×10^{-8}	1.95×10^{-7}	1.95×10^{-7}

Table S4. Evaluation of robustness of analyses. Evaluation of the impact a 10% or 20% deviation in the number of B1-8 cells has on calculated binding equilibria across the vector of 80,000 values, distributed with 10,000 random values across each of the 8 logarithmic intervals from 10^{-11} to 10^{-3}.
