# Supplementary material for: Antigen footprint governs activation of the B cell receptor
Source: Nat Commun. 2023 Feb 22;14:976. doi: 10.1038/s41467-023-36672-0 (PMC9947222; doi:10.1038/s41467-023-36672-0)
Supplement: Supplementary file 3 — Reporting Summary [file 41467_2023_36672_MOESM3_ESM.pdf]

nature portfolio

Corresponding author(s): Søren Egedal Degn

Last updated by author(s): Jan 26, 2023

Reporting Summary

Nature Portfolio wishes to improve the reproducibility of the work that we publish. This form provides structure for consistency and transparency in reporting. For further information on Nature Portfolio policies, see our Editorial Policies and the Editorial Policy Checklist.

Statistics

For all statistical analyses, confirm that the following items are present in the figure legend, table legend, main text, or Methods section.

n/a	Confirmed	
☐	☒	The exact sample size (*n*) for each experimental group/condition, given as a discrete number and unit of measurement
☐	☒	A statement on whether measurements were taken from distinct samples or whether the same sample was measured repeatedly
☐	☒	The statistical test(s) used AND whether they are one- or two-sided<br>*Only common tests should be described solely by name; describe more complex techniques in the Methods section.*
☒	☐	A description of all covariates tested
☐	☒	A description of any assumptions or corrections, such as tests of normality and adjustment for multiple comparisons
☐	☒	A full description of the statistical parameters including central tendency (e.g. means) or other basic estimates (e.g. regression coefficient) AND variation (e.g. standard deviation) or associated estimates of uncertainty (e.g. confidence intervals)
☐	☒	For null hypothesis testing, the test statistic (e.g. *F*, *t*, *r*) with confidence intervals, effect sizes, degrees of freedom and *P* value noted<br>*Give P values as exact values whenever suitable.*
☒	☐	For Bayesian analysis, information on the choice of priors and Markov chain Monte Carlo settings
☒	☐	For hierarchical and complex designs, identification of the appropriate level for tests and full reporting of outcomes
☒	☐	Estimates of effect sizes (e.g. Cohen's *d*, Pearson's *r*), indicating how they were calculated

Our web collection on statistics for biologists contains articles on many of the points above.

Software and code

Policy information about availability of computer code

Data collection	Agilent NovoExpress v. 1.5.0, BD FACSDiva v. 8.0.2, AMNIS INSPIRE v. 200.1.620.0, ZEN v. 2 (Blue edition)
Data analysis	FIDA software suite v. 2.04, AMNIS IDEAS v. 6.2.64.0, R v. 4.0.5, Python v. 3.10.2, MATLAB 2012A, EVILFIT v. 3, FlowJO v. 10.8.0, FCSExpress v. 7, Fiji (ImageJ2, v. 2.3.0), Picasso v. 0.2.7, ChemDraw Professional v. 17.1.<br>Custom code associated with the article is publicly available on GitHub (https://github.com/jungmannlab/picasso). The R script used for binding curve modeling (Figures S16 and associated tables) is available on Zenodo via the DataDryad DOI provided below.

For manuscripts utilizing custom algorithms or software that are central to the research but not yet described in published literature, software must be made available to editors and reviewers. We strongly encourage code deposition in a community repository (e.g. GitHub). See the Nature Portfolio guidelines for submitting code & software for further information.

Data

Policy information about availability of data

All manuscripts must include a data availability statement. This statement should provide the following information, where applicable:

- Accession codes, unique identifiers, or web links for publicly available datasets
- A description of any restrictions on data availability
- For clinical datasets or third party data, please ensure that the statement adheres to our policy

The datasets generated during the current study are available from Dryad at: DOI https://doi.org/10.5061/dryad.bg79cnpfb.

Field-specific reporting

Please select the one below that is the best fit for your research. If you are not sure, read the appropriate sections before making your selection.

☒ Life sciences ☐ Behavioural & social sciences ☐ Ecological, evolutionary & environmental sciences

For a reference copy of the document with all sections, see nature.com/documents/nr-reporting-summary-flat.pdf

Life sciences study design

All studies must disclose on these points even when the disclosure is negative.

Sample size	Sample size calculations were not performed a priori. Sample sizes were chosen based on the typical number of replicates reported for similar studies. A posteori, robust and statistically significant differences between groups across experiments indicated sufficient power based on the chosen sample sizes, with biologically meaningful differences in the experimental readouts between groups.
Data exclusions	No data were excluded from the analyses.
Replication	All experiments were repeated three times independently unless otherwise noted. All attempts at replication were successful.
Randomization	Randomization was not relevant to this study because cells used in experiments were derived from inbred transgenic animals on C57BL/6J background, differing only in absence (C57BL/6J) or presence and nature of BCR knock-in (B1-8hi and B1-8i). Hence, experimental group assignment was determined directly by the appropriate genotype for each group.
Blinding	No blinding was performed. For Figures 1, 2, 5 and associated supplementary figures, blinding was irrelevant because all analyses were performed using automated algorithms. For Figure 3, the sample identity was inherently represented by the read-out, precluding blinding during gating. For Figures 3, 6 and 7 blinding was not possible for practical considerations, as this was a single experimenter setup, in which samples were titrated and controls were applied in the highest assayed concentration, necessitating experimenter identification of samples and controls. All read-outs were quantitative, rather than qualitative, minimizing the risk of experimenter bias in interpreting the results.

Reporting for specific materials, systems and methods

We require information from authors about some types of materials, experimental systems and methods used in many studies. Here, indicate whether each material, system or method listed is relevant to your study. If you are not sure if a list item applies to your research, read the appropriate section before selecting a response.

Materials & experimental systems

n/a	Involved in the study	
☐	☒	Antibodies
☒	☐	Eukaryotic cell lines
☒	☐	Palaeontology and archaeology
☐	☒	Animals and other organisms
☒	☐	Human research participants
☒	☐	Clinical data
☒	☐	Dual use research of concern

Methods

n/a	Involved in the study	
☒	☐	ChIP-seq
☐	☒	Flow cytometry
☒	☐	MRI-based neuroimaging

Antibodies

Antibodies used

Purified Rat Anti-Mouse CD16/CD32 (Mouse BD Fc Block™), Clone 2.4G2, Catalog No.: 553142. Lot. 1293770
Pacific Blue Rat Anti-Mouse CD45R/B220 (BD), Clone RA3-6B2, Catalog No.: 558108. Lot. 8053605
FITC Rat Anti-Mouse CD45R/B220 (BD), Clone RA3-6B2, Catalog No.: 553088. Lot. 6349700
FITC Hamster Anti-Mouse CD3e (BD), Clone 145-2C11, Catalog No.: 553061. Lot. 6053673
PerCP/Cy5.5 anti-mouse CD8a (Biolegend), Clone 53-6.7, Catalog No.: 100734. Lot. B339471
PerCP Rat Anti-Mouse CD4 (BD), Clone RM4-5, Catalog No.: 553052. Lot. 0142773
FITC Mouse Anti-Mouse IgM(a) (BD), Clone DS-1, Catalog No.: 553516. Lot. 9015619
BV650 Mouse Anti-Mouse IgM(b) (BD), Clone AF6-78, Catalog No.: 742346. Lot. 0135903
Goat Anti-Mouse IgG(H+L), Unconjugated, highly cross-adsorbed and biotinylated (Invitrogen), Catalog No.: A16080. Lot. 50-66-042816
Purified Rat Anti-Mouse Ig λ1, λ2 & λ3 Light Chain (BD), Clone R26-46, Catalog No.: 553432. Lot. 8243749
Recombinant Mouse Monoclonal Anti-hapten 4-hydroxy-3-nitrophenyl acetyl (NP) antibody (Abcam), clone B1-8, Catalog No.: ab206523. Lot. GR3248401
Purified Mouse Anti-Mouse I-A[b] (BD), Clone AF6-120.1, Catalog No.: 553549. Lot. 5274525
PE Rat Anti-Mouse CD45R/B220 (BD), Clone RA3-6B2, Catalog No.: 553090. Lot. 6259939
Alexa Fluor 700 Rat anti-Mouse CD19 (BD), Clone 1D3, Catalog No.: 557958. Lot. 8340797

APC anti-mouse CD79b (Igβ) (BioLegend), Clone HM79-12, Catalog No.: 132807. Lot. B283042
Alexa Flour 488 anti-mouse IgD (BioLegend), Clone 11-26c.2a, Catalog No.: 405718. Lot. B323280

Validation

553142: Application: Blocking, Flow cytometry (Routinely Tested), Immunohistochemistry-frozen (Tested During Development), Immunoprecipitation (Reported). Reactivity: Mouse (QC Testing). Ref: https://www.bdbiosciences.com/en-us/products/reagents/flow-cytometry-reagents/research-reagents/single-color-antibodies-ruo/purified-rat-anti-mouse-cd16-cd32-mouse-bd-fc-block.553142
558108: Application: Flow cytometry (Routinely Tested). Reactivity: Mouse (QC Testing), Human (Reported). Ref: https://www.bdbiosciences.com/en-us/products/reagents/flow-cytometry-reagents/research-reagents/single-color-antibodies-ruo/pacific-blue-rat-anti-mouse-cd45r.558108
553088: Application: Flow cytometry (Routinely Tested), Immunofluorescence (Reported). Reactivity: Mouse (QC Testing), Human (Reported). Ref: https://www.bdbiosciences.com/en-us/products/reagents/flow-cytometry-reagents/research-reagents/single-color-antibodies-ruo/fitc-rat-anti-mouse-cd45r-b220.553088
553061: Application: Flow cytometry (Routinely Tested), Fluorescence microscopy (Reported). Reactivity: Mouse (QC Testing). Ref: https://www.bdbiosciences.com/en-us/products/reagents/flow-cytometry-reagents/research-reagents/single-color-antibodies-ruo/fitc-hamster-anti-mouse-cd3e.553061
100734: Application: FC - Quality tested. Verified Reactivity: Mouse. Ref: https://www.biolegend.com/ja-jp/neuroscience-1/percp-cyanine5-5-anti-mouse-cd8a-antibody-4255
553052: Application: Flow cytometry (Routinely Tested): Reactivity: Mouse (QC Testing). Ref: https://www.bdbiosciences.com/en-us/products/reagents/flow-cytometry-reagents/research-reagents/single-color-antibodies-ruo/percp-rat-anti-mouse-cd4.553052
553516: Application: Flow cytometry (Routinely Tested), Immunofluorescence (Tested During Development). Reactivity: Mouse (QC Testing). Ref: https://www.bdbiosciences.com/en-us/products/reagents/flow-cytometry-reagents/research-reagents/single-color-antibodies-ruo/fitc-mouse-anti-mouse-igm-a.553516
742346: Application: Flow cytometry (Qualified). Reactivity: Mouse (Tested in Development). Ref: https://www.bdbiosciences.com/en-us/products/reagents/flow-cytometry-reagents/research-reagents/single-color-antibodies-ruo/bv650-mouse-anti-mouse-igm-b.742346
A16080: Application: ELISA. Reactivity: Mouse. Ref: https://www.citeab.com/antibodies/2401033-a16080-goat-anti-mouse-igg-h-l-highly-cross-adsorb?utm_campaign=Widget+All+Citations&utm_medium=Widget&utm_source=Thermo+Secondary+Abs+Widget&utm_term=Invitrogen+Antibodies
553432: Application: ELISA (Routinely Tested). Reactivity: Mouse (QC Testing). Ref: https://www.bdbiosciences.com/en-us/products/reagents/immunoassay-reagents/elisa/purified-rat-anti-mouse-ig-1-2-3-light-chain.553432
ab206523: Application: WB, Flow Cyt. Reacts with: Species independent. Ref: https://www.abcam.com/hapten-4-hydroxy-3-nitrophenyl-acetyl-np-antibody-b1-8-ab206523.html
553549: Application: Flow cytometry (Routinely Tested). Reactivity: Mouse (QC Testing). Ref: https://www.bdbiosciences.com/en-us/products/reagents/flow-cytometry-reagents/research-reagents/single-color-antibodies-ruo/purified-mouse-anti-mouse-i-a-b.553549
553090: Application: Flow cytometry (Routinely Tested). Reactivity: Mouse (QC Testing), Human (Reported). Ref: https://www.bdbiosciences.com/en-us/products/reagents/flow-cytometry-reagents/research-reagents/single-color-antibodies-ruo/pe-rat-anti-mouse-cd45r-b220.553090
557958: Application: Flow cytometry (Routinely Tested). Reactivity: Mouse (QC Testing). Ref: https://www.bdbiosciences.com/en-be/products/reagents/flow-cytometry-reagents/research-reagents/single-color-antibodies-ruo/alexa-fluor-700-rat-anti-mouse-cd19.557958
132807: Application: FC - Quality tested. Verified Reactivity: Mouse. Ref: https://www.biolegend.com/it-it/products/apc-anti-mouse-cd79b-igbeta-antibody-13911
405718: Application: FC - Quality tested, SB - Reported in the literature. Verified Reactivity: Mouse. Ref: https://www.biolegend.com/it-it/products/alexa-fluor-488-anti-mouse-igd-7092

Animals and other organisms

Policy information about studies involving animals; ARRIVE guidelines recommended for reporting animal research

Laboratory animals

B1-8hi (B6.129P2-Ptprca Ightm1Mnz/J); B1-8i (B6.129P2(C)-Ightm2Cgn/J) crossed with Jk-knock-out (B6.129Sv(C)-Igkjctm1/J); C57BL/6JRj. Both male and female mice were used in experiments. Mice were used from 6-30 weeks of age. The mice were maintained in our SPF vivarium at the Department of Biomedicine, Aarhus University, in IVCs on a standard 12-hr light/dark cycle, with standard chow and water ad libitum, at ambient room temperature (20-22 degrees C) and ambient humidity.

Wild animals

The study did not involve wild animals.

Field-collected samples

The study did not involve samples collected from the field.

Ethics oversight

The Danish Animal Experiments Inspectorate.

Note that full information on the approval of the study protocol must also be provided in the manuscript.

Flow Cytometry

Plots

Confirm that:

☒ The axis labels state the marker and fluorochrome used (e.g. CD4-FITC).

☒ The axis scales are clearly visible. Include numbers along axes only for bottom left plot of group (a 'group' is an analysis of identical markers).

☒ All plots are contour plots with outliers or pseudocolor plots.

☒ A numerical value for number of cells or percentage (with statistics) is provided.

Methodology

Sample preparation	Spleens were harvested from freshly euthanized mice, transferred into ice-cold FACS buffer (PBS, 2% FCS, 2 mM EDTA), and mechanically dissociated using a plunger from a 10 ml syringe in a 70 µm cell strainer, followed by rinsing with ice-cold FACS buffer. The cells were recovered by centrifugation at 200 g for 5 minutes at 4°C. Erythrocytes were lysed by adding RBC lysis buffer (155 mM NH4Cl, 12 mM, NaHCO3, 0.1 mM EDTA) followed by incubation at room temperature for 2-3 minutes. Ice-cold FACS buffer was added and the splenocytes were recovered by centrifugation as before and finally resuspended in ice-cold FACS buffer before proceding with B cell purification and/or staining and analysis.
Instrument	Calcium flux and flow cytometry: NovoCyte Quanteon 4025 (Agilent). Imaging flow cytometry: ImageStreamX MkII (Amnis, Luminex Corporation) at x60 magnification.
Software	Agilent NovoExpress v. 1.5.0, AMNIS INSPIRE v. 200.1.620.0, FlowJO v. 10.8.0, FCSExpress v. 7
Cell population abundance	No sorting was performed.
Gating strategy	Gating strategy provided in Figure S9 for purification strategy. Gating for selection of B1-8hi population for calcium flux analyses provided in Figure 4. Gating for selection of HJ-NP/NIP binders provided in Figures 3 and S10.

☒ Tick this box to confirm that a figure exemplifying the gating strategy is provided in the Supplementary Information.
